# NEUROFILAMENT LIGHT CHAIN (NFL) AND GENERAL COGNITIVE ABILITY IN ADULTS APPROACHING MIDLIFE

**DOI:** 10.1093/geroni/igac059.2911

**Published:** 2022-12-20

**Authors:** Chandra Reynolds, Andrew Smolen, Christopher Link, Donald Evans, Ryan Bruellman, Luke Evans, Sally Wadsworth

**Affiliations:** University of California, Riverside, Riverside, California, United States; University of Colorado Boulder, Boulder, Colorado, United States; University of Colorado Boulder, Boulder, Colorado, United States; University of Colorado Boulder, Boulder, Colorado, United States; University of California, Riverside, Riverside, California, United States; University of Colorado Boulder, Boulder, Colorado, United States; University of Colorado Boulder, Boulder, Colorado, United States

## Abstract

Neurofilament light chain (NfL) is a biomarker indexing axonal integrity where small NfL variations may be associated with cognitive performance in early adulthood and high values associated with neurodegenerative disorders such as Alzheimer’s disease. In the Colorado Adoption/Twin Study of Lifespan behavioral development and cognitive aging (CATSLife1) individuals were tested at 28–49 years (M=33.1, SD=4.9). Quanterix Simoa assays of plasma NfL (pNfL) were measured in duplicate, and we included values for 1159 individuals where 1098 had available general cognitive ability scores and sociodemographic covariates. Unadjusted NfL values were consistent with other studies of early-mid adulthood (M = 5.9, SD = 3.1, range = 1.14 – 40.1 pg/mL) and 6% showed values outside expected normal reference limits (>10 pg/mL). After adjusting for technical covariates and skew, higher natural log-transformed pNfL was associated with age (r = 0.27) and female sex (r = 0.07). Moreover, adjusting for sociodemographic covariates, higher pNfL was associated with lower general cognitive ability (GCA) (r = -.06), where associations were more pronounced above the mean pNfL value (r = -.08). Multi-level regression analyses suggested that GCA-NfL associations were modified by age, whereby the worse performance was observed at higher ages and pNfL values (p <= 0.03), accounting for sibling relatedness and sociodemographic covariates. We observed small negative associations of higher plasma NfL and lower cognitive performance, where associations may become magnified with increasing age in early- to mid-adulthood.

